# A comparison of two techniques to identify the sex of the eastern blue‐tongue skink (*Tiliqua scincoides scincoides*)

**DOI:** 10.1111/avj.13170

**Published:** 2022-05-12

**Authors:** A. McKenzie, T. Li, B. Doneley

**Affiliations:** ^1^ School of Veterinary Science University of Queensland Gatton Queensland; ^2^ School of Agriculture and Food Sciences The University of Queensland Gatton Queensland Australia

**Keywords:** contrast radiography, eastern blue‐tongued, hemeclitores, hemepenes, morphometrics, sex identification, skink

## Abstract

**Introduction:**

The eastern blue‐tongued skink (*Tiliqua scincoides scincoides*), native to eastern Australia, is commonly kept as both a pet and for breeding. As a sexually monomorphic species, it is important to develop reliable techniques for sex identification, both for breeding and health purposes. Numerous techniques have been developed for the identification of sex in other reptile species but, other than possibly morphometric analysis, none have proven to be reliable in this species. Two techniques showing promise are contrast radiography of the hemepenes/hemeclitores, and morphometrical analysis. This study looks at both techniques and compares them for accuracy.

**Methods and Materials:**

Twenty captive eastern blue‐tongued skinks (of known sex) were sedated, contrast radiography of their hemepenes /hemeclitores was performed, and physical measurements were taken for morphometric analysis. The radiographs were examined by a panel of three researchers (blinded to the known sex) to identify sex. The morphometric data were statistically analysed, following a previously published methodology, and the individual sex identified. Again, the researchers were blinded to the known sex.

**Results:**

The contrast radiography technique was 100% accurate in correctly identifying the sex of all the skinks. Morphometric analysis was, by contrast, only 70% accurate.

**Conclusion and Clinical Relevance:**

Physical differences between wild and captive skinks, as well as different environmental and nutritional factors, may have contributed to the lower accuracy of morphometric analysis in identifying the sex of eastern blue‐tongued skinks. While contrast radiography was more accurate, the need for specialised equipment may render this technique impractical for field researchers, but more suitable for owned animals. More research is needed to assess the impact of captivity on eastern blue‐tongued skinks' physical morphometrics.

**Abbreviations**
H: widest point of the head
H/SVL%: head width divided by snout‐vent length
H/T%: head width divided by trunk length.
HL: head length from the rostral tip of the head to the back of the ears
SVL: snout‐vent length from the rostral tip of the head to the middle of the vent
T: trunk length from the middle of the forelimbs to the middle of the hindlimbs
TBL: total body length from the rostral tip of the head to the distal tip of the tail
TW: tail width at the widest point of the proximal tail

## Introduction

**Figure 1:**
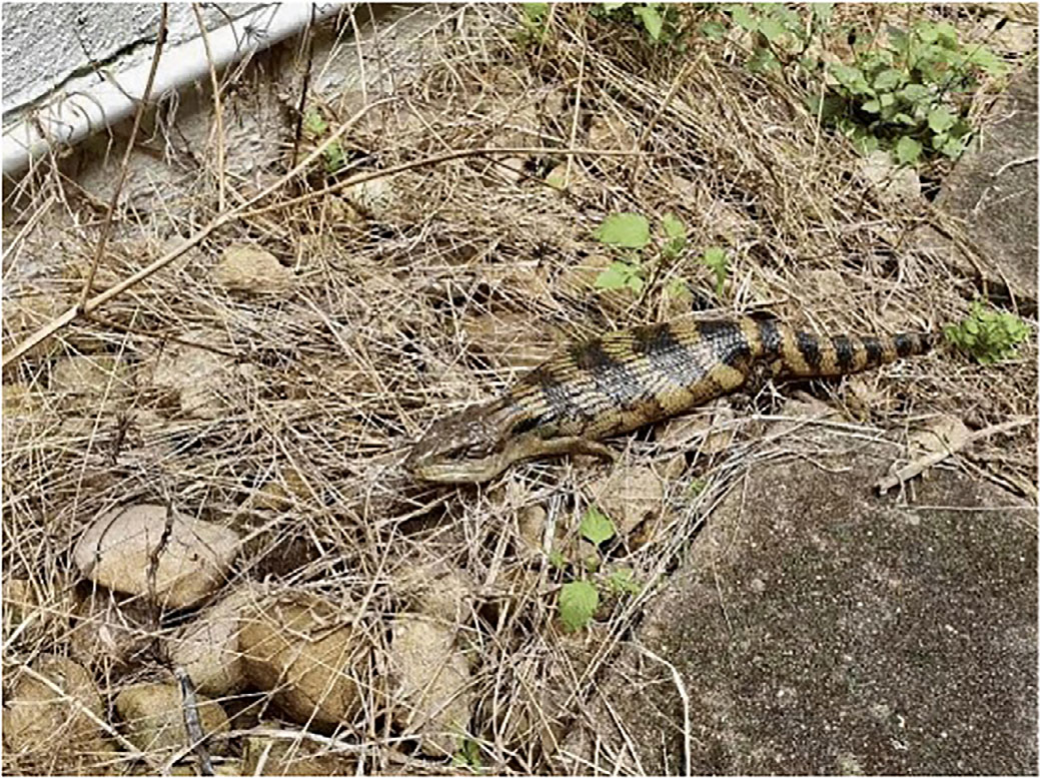
The eastern blue‐tongued skink (*Tiliqua scincoides scincoides*)

The blue‐tongued skinks are members of the skink family (*Scincidae*), genus *Tiliqua*, with eight species; six are found in Australia and two in New Guinea and Indonesia. As such, this genus has evolved to live in vastly differing environments with an array of nutritional pressures.^1^ They are one of the largest terrestrial skinks in the skink family, with an average body length of 20–25 cm and an average weight of 200–440 grams.^2^ The eastern blue‐tongued skink (*Tiliqua scincoides scincoides*) (Figure 1) is native to eastern Australia, from north‐eastern Queensland, through central and eastern New South Wales, to Victoria. It is considered to be one of the more common skinks in eastern Australia, as they have adapted well to city and urban life. It is an increasingly popular exotic pet due to its size, docile nature, low‐maintenance of care^3^ and longevity, with many living for over 20 years in captivity.^4^, ^5^ Many herpetoculturists keep this species to breed for colour mutations and for the pet market.^4^

**Figure 2:**
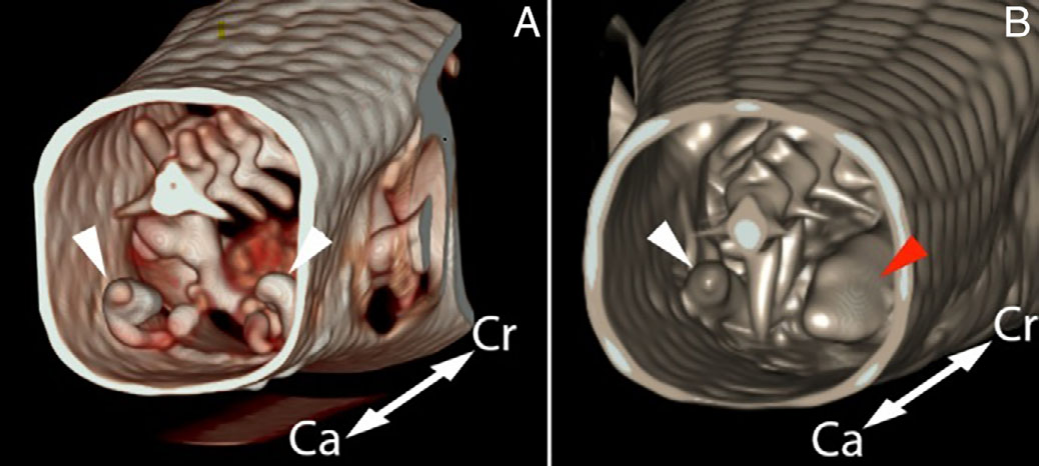
Three‐dimensional reconstruction of a computed tomography scan of the tail base of a male (a) and a female (b) eastern blue‐tongued skink. The male hemepenes (white arrowheads) are seen as a spiralling structure, compared with the female straight hemeclitores (white and red arrowheads). The female's right hemiclitoris (red arrowhead) is significantly dilated with contrast media. (ca, caudal; Cr, cranial).

They are sexually monomorphic, that is, both sexes are similar in their physical appearance. There is no difference in colouration,^1^ they exhibit only subtle (or no) physical sexual dimorphism and their reproductive organs are internal. Skinks have paired gonads located within the coelom, connected to the cloaca by the paired oviducts or vas deferens. In both male and female skinks, paired hollow pockets terminating in a long retractor muscle can be found on both sides of the cloaca, opening into the caudal cloaca sulcus and directed caudally (Figure 2). In males, these pockets are known as the hemepenes; it is a wide, spiralling structure that engorges with blood and everts during copulation to insert into the female cloaca. Only one hemepenes everts during intromission. Semen, ejaculated into the cloaca, runs down the seminal groove of the hemepenes and into the female's cloaca. The female's hemiclitores is physically homologous to the hemepenes but is morphologically different in length, size and lumen shape.^6^ Like the hemepenes, it is easily eversible, but its function is not fully understood, that is, it is unclear whether it is actively everted by the female during copulatory adjustment, and/or whether it stimulates the male's hemipenes during intromission.^6^, ^7^

Other than the obvious need for sex identification when pairing male and female skinks for breeding, there are many sex‐based diseases and behaviours, such as territorial aggression, follicle retention and dystocia, which can be difficult to diagnose or prevent if the sex of the animal is unknown.^6^, ^8^ This can be particularly challenging in sexually monomorphic species, as they have no obvious external differentiating characteristics between sexes^.^ ^7^, ^9^, ^10^ A reliable technique for sex identification of blue‐tongued skinks is an essential tool for managing the health and captive breeding of this species.

Techniques of sex identification in most reptiles include morphometric analysis, probing the hemipenal pocket, hemipenal eversion, coelioscopy, ultrasound and radiography, with different techniques being preferred for different species.^11^ Previously, there have been three common and simple techniques of sex identification for monomorphic skink species.

Probing the depth of the hemipenes/hemeclitores^11^, ^12^, ^13^ has been found to be unreliable in blue‐tongued skinks with little correlation between depth of the structure and the skink's sex. (Doneley, personal observation). The hemipenes can be everted (“popped”) by holding pressure on the ventral tail base; however, this is often unreliable in individuals with muscular tails and care must be taken with species with autotomy, such as skinks.^12^ This technique can also be damaging to the hemepenes, and failure to evert the hemepenes does not guarantee the skink is female.^14^ Finally, ultrasonography of the internal gonads is useful in many skink species, and Roberts et al^15^ claimed 90% accuracy when using a 7.5 MHz convex array probe while the skink was partially submerged in a water bath. However, Doneley (personal observation) has found it to be difficult and unreliable in blue‐tongued skinks due to the presence of impenetrable osteoderms in the skin.

Two other techniques for sex identification in eastern blue‐tongues have been described. Mallet^16^ modified a technique developed by Di Ianni^7^ using contrast media to outline the hemepenes/hemeclitores on radiographs, while Phillips et al^17^ used morphometrics to distinguish between the sexes of this species. Both techniques claim a high level of accuracy, but the morphometric technique does not appear to be consistently repeatable (Doneley, personal observation). Comparing these two techniques offers researchers, veterinarians and herpetoculturists guidance on the accuracy and practicality of sex identification in the eastern blue‐tongued skink.

## Methods and materials

This research was conducted with the approval of the University of Queensland Animal Ethics Committee (AEC Approval Number: SVS/387/19, dated 15/10/2019).

### Animals

Twenty captive‐bred eastern blue‐tongued skinks were collected from two experienced hobbyist breeders and from the University of Queensland's research collection housed at Gatton, Queensland; Skinks #1–6 and #12–20 were housed in outdoor enclosures mimicking a wild environment, and skinks #7–11 were housed in indoor glass enclosures with both a heat lamp and UVB lamp. The husbandry of all animals in the study was assessed and determined by the researchers to be suitable for this species. The animals were housed by the researchers for 1 day only, and then returned to their owners.

Eighteen of the skinks were confirmed to be adults via owner breeding records, and measurement of snout‐vent length and head width compared with Shea. Only two sub‐adults (bred by the owners) were used in the study; as they were similar in size and weight to the adult skinks, they were included with the adult population for the study. The authors were blinded to the known sex until after the morphometric data were collected and contrast radiography performed.

### Limitations

Only eastern blue‐tongued skinks were used in this study; other *Tiliqua* species were not used. Only adult or sub‐adult animals were used; the authors cannot comment on the accuracy of either technique in juveniles. Only captive skinks were used; therefore, the effects of husbandry and diet were not compared with wild skinks.

As this study's sample size was heavily influenced by the availability of skinks, the lack of difference within this study is possibly from a lack of statistical power.

### Methodology

To ensure accurate measurements and radiographs were taken, each skink was sedated with 10 mg/kg alfaxalone (Alfaxan CD, Jurox, NSW Australia) intramuscularly, into either the triceps or biceps muscle. Once the procedures were completed, each skink was placed back into a heated container and monitored to ensure quick and safe recovery.

**Figure 3:**
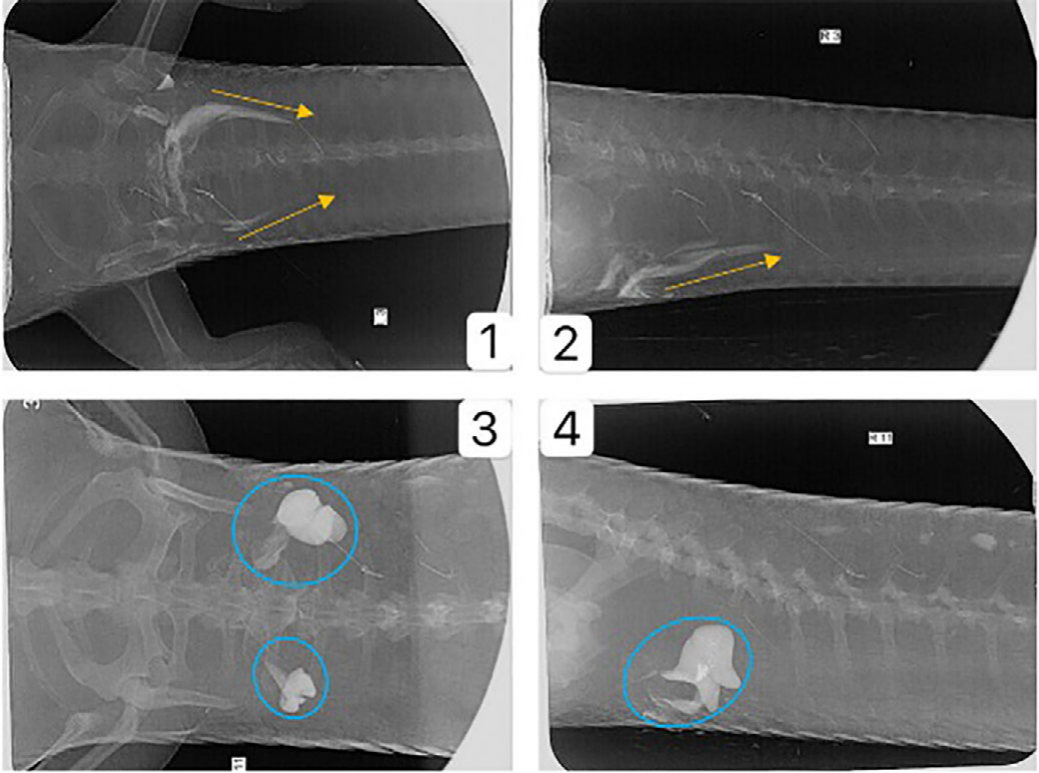
Contrast radiographs of the tail base of a male and a female eastern blue‐tongued skink. 1) Dorvoventral view of a female; 2) lateral view of a female; 3) dorsoventral view of a male; 4) lateral view of a male. The difference between the coiled hemepenes and the straight hemeclitores is clear.

Each skink was gently restrained by hand and held upright at an angle of 45° with its tail directed towards the operator. A 22 g intravenous canula (with the stylet removed) was inserted into the hemepenal/hemeclitoral opening. Once the catheter had reached its maximum depth, 1 ml of iohexal (Omnipaque, GE Healthcare Australia, Parramatta NSW, Australia) was introduced into the pocket as the catheter was slowly withdrawn, filling the pocket. Excess contrast material leaking from the pocket was wiped away with a paper towel. The procedure was then repeated on the opposite side. The skink was then placed into a transparent plastic tube to assist with restraint while the radiographs were taken using a dental Xray machine (iM3 Revolution 4 DC, iM3 Australia) with the following settings: kV 60 mA 7.0, exposure time 0.06 s, collimating on the base of the tail. Lateral and dorsoventral views of each were taken. The radiographs were then independently assessed by a panel of three researchers and the sex recorded. Skinks with a shortened spiralled structure highlighted by contrast were classified as male, while female hemiclitores were highlighted as long, straight structures. (Figure 3) No adverse effects caused by the contrast media were noted in any lizards after the study.

**Figure 4:**
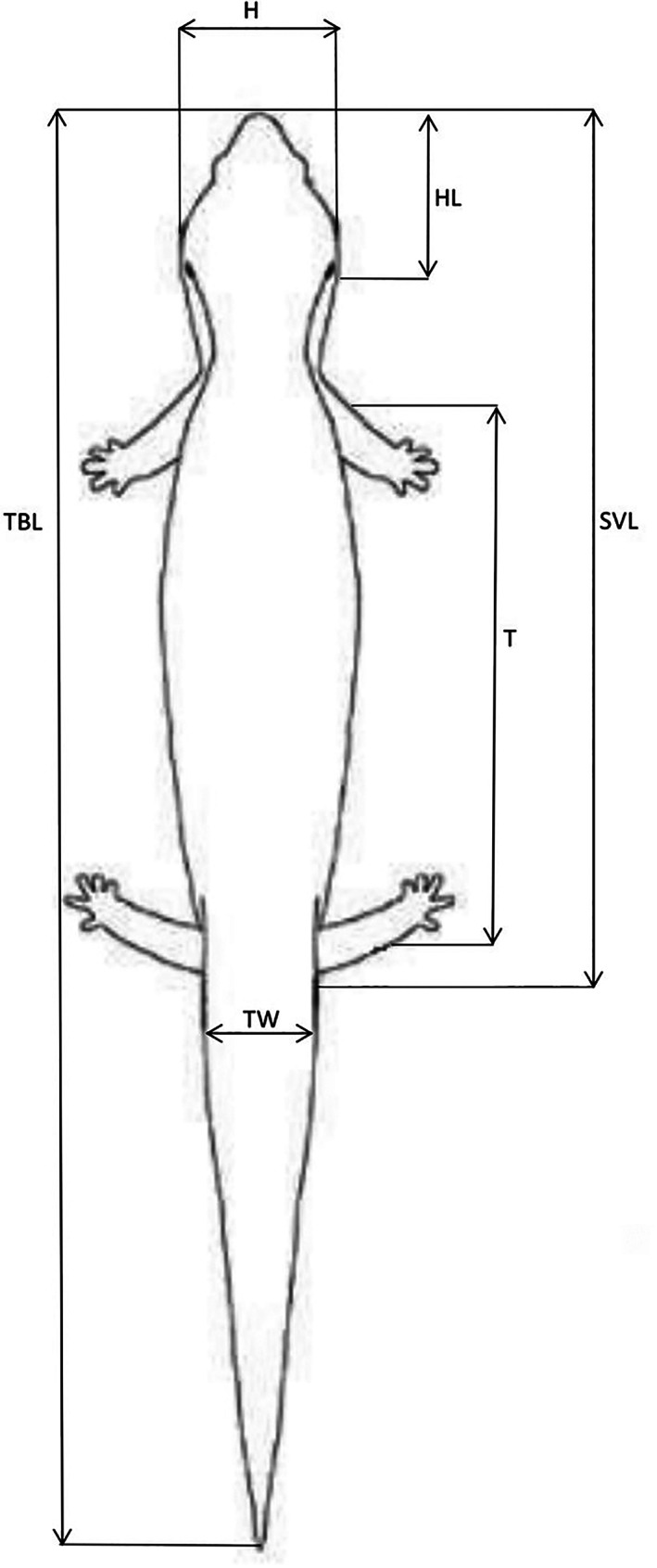
Measurements taken for morphometric analysis. H, widest point of the head; HL, head length from the rostral tip of the head to the back of the ears; SVL, snout‐vent length from the rostral tip of the head to the middle of the vent; T, trunk length from the middle of the forelimbs to the middle of the hindlimbs; TBL, total body length from the rostral tip of the head to the distal tip of the tail; TW, tail width at the widest point of the proximal tail.

Following the technique described in Phillips et al,^17^ each skink was then weighed, and any important physical features were noted (e.g., injuries or missing tail). Using digital callipers, a plastic 30 cm ruler and a tape measure, measurements were taken at the widest point of the head (H); head length from the rostral tip of the head to the back of the ears (HL); snout‐vent length from the rostral tip of the head to the middle of the vent (SVL); trunk length from the middle of the forelimbs to the middle of the hindlimbs (T); total body length from the rostral tip of the head to the distal tip of the tail (TBL); and tail width at the widest point of the proximal tail (TW). Measurements were taken in millimetres to the second decimal point. (Figure 4)

### Morphometric statistical analysis

Following the previously published method, the H/SVL% (head width over snout‐vent length) and H/T% (head width over trunk length) ratios were calculated for each skink, and they were categorised into male or female using the confidence intervals provided by Phillips et al. These results were compared against the skinks' known sex to determine the accuracy of this method. In those skinks that fell in‐between the confidence intervals provided by Phillips et al, a mean was calculated between the lowest male interval, and the highest female interval. When the skinks' individual ratio was above this mean value, the skink was categorised as male. Conversely, if the skinks' individual ratio was below this mean value, the skink was categorised as female. Weight was not considered in the data analysis to avoid varying body condition scores affecting the outcome.

### Formal statistical analysis was performed in Rstudio

First, a series of one‐way t‐tests were performed for each individual measurement and the H/SVL% and H/T% ratios, to create a new set of confidence intervals specific to this study. Then, a series of univariate logistic regressions were conducted to assess if any morphometric variables were an accurate predictor of sex, with female included as the reference category of 0. Odds ratios were calculated for each model. These logistic regression analyses, and the following analyses, used each skink's confirmed sex (from owners' breeding records and contrast radiography) as the dependent variable to investigate the association of morphometric measurements with the skinks true sex.

A series of one‐way ANOVAs were then performed to determine the potential effect of enclosure type on the size of the skinks; enclosure type was included as the dependent variable and head width, head length and tail width were each included as independent variables.

Finally, a series of two‐way ANOVAs were performed to investigate the potential confounding effect of sex on the differences found in enclosure type as there was a higher proportion of females in the outdoor enclosures, and a higher proportion of males in the indoor enclosures. Here, sex was included as the second independent variable alongside head width, head length and tail width. Again, enclosure type was included as the dependent variable.

## Results

### Contrast radiography

The sex of all 20 eastern blue‐tongued skinks was able to be definitively identified using contrast radiography, with uniform consensus between the members of the panel and 100% correlation with the known sex.

### Morphometric analysis

Using confidence intervals provided by Phillips et al, the sex of 70% of the skinks in this study were correctly identified. Overall, the male skinks had smaller head widths and trunk lengths, and more variable snout‐vent lengths than those found in the study by Phillips et al.^17^ The female skinks in this study were also overall smaller in size.

The H/SVL% and H/T% ratios for 7 of the 14 correctly identified skinks did not fit within the confidence intervals provided by Phillips et al and were adjusted to fit one of the sexes as described above. For example: Skink 2 had an H/SVL% ratio of 14.36 and an H/T% ratio of 22.27. The mean between male and female confidence intervals by Phillips et al^17^ for H/SVL% ratio is 14.6, and H/T% ratio is 22.57; therefore skink 2 was closer to the female intervals and identified as a female. This ‘female’ was then confirmed through owner records and contrast radiography to be a male. Without this adjustment, the sex of only 7 of the 20 skinks were correctly identified (35%).

Furthermore, some skinks in this study only fitted into one ratio interval. Skink 8 and skink 12 share the same SVL and H measurement, and thus the same H/SVL% of 14.4% identifying them as females. However, as skink 8's trunk length was longer, its H/T% (20.5%) was lower than skink 12's (22.2%) and it fitted well into the female interval. Both skinks were then confirmed to be males.

**Figure 5:**
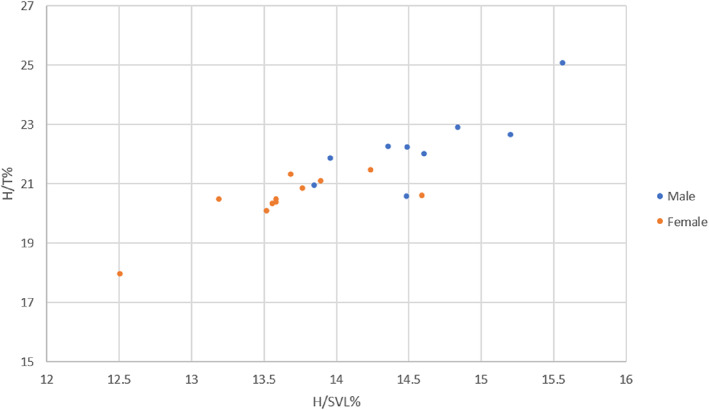
Comparison of all H/SVL% and H/T% ratios between sexes. There is a strong, positive, linear association between the two ratios for both sexes, however there is moderate overlap between the lower range males and higher range females.

Calculating new 95% confidence intervals for the H/SVL% and H/T% ratios (Table 1.) resulted in separate value ranges for both the female and male skinks. The male skinks had higher values; however, these new confidence intervals were close in range due to overlap in low male ratio values and high female ratios (Figure 5). The female skinks had very similar confidence intervals for the H/SVL% and H/T% ratios to those reported in Phillips et al,^17^ and the male skinks had significantly lower value ranges than those reported in Phillips et al.^17^ Ultimately, the odds ratio calculated for the H/SVL% ratio and H/T% ratio in this study indicated that for every 1% increase in these ratios, the skinks are 49‐fold and 14‐fold respectively, more likely to be a male (Table 2.). The only significant differences (*P* ≤ 0.05) found in this study were between the males and females H/SVL% (*p* = 0.017) and H/T% (*p* = 0.019) ratios, and between enclosures; indicating those in the indoor enclosures had both longer (*p* = 0.005) and wider heads (*p* = 0.034) than those in outdoor enclosures.

**Table 1:** Confidence intervals for H/SVL% and H/T% ratio's for the female and male skinks

Lizard	Weight (g)	SVL (mm)	H (mm)	HL (mm)	T (mm)	TBL (mm)	TW (mm)	H/SVL% ratio	H/T% ratio	Estimated sex using Phillips (2016) ratio's	Confirmed known sex	Estimated morphometric data match confirmed sex
*Outdoor enclosure*												
Lizard 1	361	283	35.39	49.7	197	409	29.2	12.5053	17.9644	Female	Female	Yes
Lizard 2	308	273	39.2	51.53	176	435	26.25	14.3589	22.2727	Female	Male	No
Lizard 3	386	255	36.3	46.48	169	396	22.4	14.2352	21.4792	Female	Female	Yes
Lizard 4	232	247	34	47.1	163	400	19.8	13.7651	20.8588	Female	Female	Yes
Lizard 5	733	315	45.95	57	223	503	34.5	14.5873	20.6053	Female	Female	Yes
Lizard 6	555	321	44.44	55.49	212	497	29.3	13.8442	20.9622	Female	Male	No
*Indoor enclosure*												
Lizard 7	418	282	43.88	61.46	175	441	29.8	15.5602	25.0742	Male	Male	Yes
Lizard 8	411	290	42	59.3	204	469	28.4	14.4827	20.5882	Female	Male	No
Lizard 9	367	284	39.45	48.86	187	415	29.39	13.8908	21.0962	Female	Female	Yes
Lizard 10	585	312	42.38	58.67	208	470	33.4	13.5833	20.375	Female	Female	Yes
Lizard 11	621	304	46.213	60.75	204	492	37.85	15.2016	22.6534	Male	Male	Yes
Lizard 12	323	290	42.02	52.05	189	444	28.38	14.4896	22.2328	Female	Male	No
*Outdoor enclosure*												
Lizard 13	228	244	36.2	45.96	158	391	24.7	14.8360	22.9113	Male	Male	Yes
Lizard 14	408	292	38.5	47.8	188	448	29.6	13.1849	20.4787	Female	Female	Yes
Lizard 15	383	281	37.98	47.86	189	445	27.63	13.5160	20.0952	Female	Female	Yes
Lizard 16	327	270	36.6	47.64	180	424	25.36	13.5555	20.3333	Female	Female	Yes
Lizard 17	292	271	37.82	51.6	173	442	26.54	13.9557	21.8612	Female	Male	No
Lizard 18	296	279	37.89	51.49	185	439	26.75	13.5806	20.4810	Female	Female	Yes
Lizard 19	409	276	40.31	55.17	183	463	28.45	14.6050	22.0273	Female	Male	No
Lizard 20	581	324	44.34	53.81	208	495	31.53	13.6851	21.3173	Female	Female	Yes

**Table 2:** Univariate logistic regression outcomes predicting skink sex

Independent variable	Estimate	Standard error	Z‐value	*p*‐value	Odds ratio
H/SVL%	3.892	1.6344	2.382	0.017	49.008
H/T%	2.688	1.150	2.338	0.019	14.702

*p*‐values reported correspond to univariate logistic regressions. All results are on 19 degrees of freedom. Female to the effect of 0.

## Discussion

### Contrast radiography

Di Ianni et al^7^ used contrast radiography for sex identification in four species of lizards, including the blue‐tonged skink, by applying a contrast media shallowly into the cloaca. This technique was 94.7% accurate, second only to those of contrast computed tomography (CT). Mallett^16^ modified Di Ianni's method by placing 1 ml of iohexal directly into the hemepenes with a syringe and catheter. The contrast medium effectively highlighted the homologous structures in the image, with spirals for males and straight for females, allowing the identification of the sex with 100% accuracy. Our study confirmed the high level of accuracy of this technique.

### Morphometric analysis

Morphometric analysis, using the technique described in Phillips et al,^17^ correctly identified the sex of only 35% skinks in this study, and this increased to 70% when adjustments were made to fit each skink into a category. In addition, the reduced distance between the 95% confidence intervals in this study and largely overlapping body and head size between sexes, combined with the significantly higher odds of a skink being male with increasing H/SVL% and H/T% than in Phillips et al,^17^ seem to contradict each other. This discrepancy in study results but apparent difference between sexes makes it difficult to determine whether this variability is due to true species‐specific sexual dimorphism, some form of geographical phenotypic plasticity (Via), or due to the effects of captivity. Phillips et al's study used wild‐caught skinks, presumably on a more natural diet, good exposure to sunlight (and therefore UVB radiation), and more exercise. Both sexes snout‐vent lengths were similar in range to the female snout‐vent lengths found in New South Wales; however, the variability of the males' snout‐vent length matched those found in Queensland.^8^ Nevertheless, there are many husbandry‐related possibilities resulting in a reduced level of sexual dimorphism experienced in this study.

Common husbandry problems such as high population density, decreased food availability or quality, or a suboptimal thermoregulatory zone may be prevalent in both indoor and outdoor enclosures. The increased head size in indoor skinks regardless of sex indicates the likelihood of the skinks being impacted from their environment. The indoor skinks may have been exposed to a higher population density, and therefore increased mating‐induced male‐to‐male combat, and increased head size over generations.^18^, ^19^ Certain food types given over generations can also impact the skinks head width and length due to their adaption to both lingual and jaw prey capture.^20^ Low food availability due to inadequate care or high competition, predator awareness, and energy diversion to reaching sexual maturity over body growth has also been found to play a part in reduced growth rates, particularly in reduced snout‐vent lengths, in lizards.^21^, ^22^, ^23^ Juveniles in particular are susceptible to excessive energy expenditure and reduced growth rates from inadequate thermoregulatory zones,^24^ particularly if they are not housed separately to adults, as they require different basking patterns.^25^ Both head size and sex have also been found to be affected by the mother's temperature throughout gestation in viviparous skinks.^26^ It is possible the indoor skinks were provided with their ideal environment year‐round, promoting their growth beyond those in the outdoor enclosures.

Ultimately, further research is needed on the impacts of feed availability and habitat types on potential eastern blue‐tongued growth due to the vastly different environmental controls, feeding habitats and competition experienced between wild and captive skinks. Brumation periods may also play a role in the development of these skinks in various enclosures, but it is an understudied phenomenon.

Future researchers are encouraged to include history on generational lineage, original source of the skinks, thorough exploration of their enclosure and to source skinks known to be from different environments, wild and captive, from across Australia. The population in this study was chosen at random, and therefore assumed to be normally and independently distributed. However, there was still an element of possible stratification due to ancestry or enclosure type that may have influenced the skinks growth due to the authors having to request admittance into the study. The original source of the skinks in this study is unknown; therefore, it is difficult to interpret the morphometric differences and similarities alongside collective data such as Shea.^8^

As this study's sample size was heavily influenced by the availability of skinks, the lack of statistical difference within this study is possibly from a lack of power. As there is evidence of morphological differences between male and female eastern blue‐tongued skinks (i.e., males have longer heads) without statistical significance supporting it, the poor post‐hoc analyses may indicate a lack of power to detect the true effects of these variables. The high standard error also indicates a poor representation of the population, as the means for these factors are highly distributed. However, a major factor limiting the accuracy of this technique is interpersonal human error. Vrdoljak et al^27^ showed that measuring morphologic geometrics in lizards can result in a large margin of interpersonal error (between 19.5% and 60%). Only one author of this study performed the morphometric measurements to ensure the technique was repeated as closely as possible between subjects, however this is a risk when implementing morphometric analysis.

The current lack of statistical power and potential impact of captivity on the sex identification of eastern blue‐tongued skinks should be further investigated with a larger balanced sample population, and multiple researchers repeating the technique. This will provide more comprehensive confidence intervals and test the true significance of the variables used in this study.

## Conclusion

This study indicates that the use of contrast radiography to identify the sex of captive eastern blue‐tongued skinks is more accurate than morphometrical analysis. However, the radiography technique requires equipment and materials that may not be readily available to researchers in the field or herpetoculturists. For these groups, morphometric analysis may be more practical and, in wild eastern blue‐tongued skinks, nearly as accurate as contrast radiography.

More research is needed in both techniques across a larger number of skinks, different age groups and species, and different husbandry and diet before widespread acceptance of either technique can be justified.

## Conflicts of interest and sources of funding

The authors declare no conflicts of interest or sources of funding for the work presented here. This project was funded by the University of Queensland (School of Veterinary Science and the School of Agriculture and Food Sciences).
